# Distal extension of palatal rugae as a limitation for donor soft tissue grafts in a Jordanian population: A cross-sectional study

**DOI:** 10.1186/s12903-021-01561-9

**Published:** 2021-04-23

**Authors:** Khalid Nazmi Said, Areej Sulaiman Abu Khalid, Fathima Fazrina Farook

**Affiliations:** 1 Department of Dentistry, Oral Health Institute, Hamad Medical Corporation, Doha, Qatar; 2 Department of Dental and Oral Health, Prince Sultan Military College of Health Sciences, Dhahran, Kingdom of Saudi Arabia; 3 College of Dentistry, King Saud Bin Abdulaziz University for Health Sciences, Riyadh, 11426 Saudi Arabia; 4 King Abdullah International Medical Research Centre, Riyadh, Saudi Arabia

**Keywords:** Palatal rugae, Jordanian population, Rugae patterns, Posterior extension

## Abstract

**Background:**

The purpose of the cross sectional study was to investigate the distal extension of the rugae area in a Jordanian (Middle Eastern) population, as an anatomical limitation influencing the surgical decision of harvesting a palatal soft tissue graft. Factors that may influence or predict the extension were also assessed.

**Methods:**

Sixty periodontally healthy participants (29 males and 31 females) were included. Maxillary alginate impressions were made and casts were poured. The measurements were highlighted from the origin of the rugae (near mid palatine raphe) to the terminal end with a sharp graphite pencil on the cast and a magnification lens was used for identification. The most posterior extension of the rugae were marked on the casts and determined by a standardized periodontal probe. The normal approximation test for binomial distribution was used to determine the proportion of the subjects with rugael extensions beyond the mesial end of the upper second premolar and fisher’s exact test for the purpose of analysis of the association of this extension with other factors.

**Results:**

In almost half (41.7%) of the sample, the rugae extended distal to the upper second premolar, 23.3% extended to the mid-palatal of the upper second premolar, and 11.7% extended to the mesial of the upper second premolar. The implication is that 90.0% of the rugae reached the upper second premolar and 78.3% extended beyond its mesial aspect. The normal approximation test performed with 95% CI with the "rugael extension proximal to the mesial end of the upper 2nd premolar" considered to be the "success" category revealed that the proportion of the subjects with rugael extensions proximal to the mesial end of the upper second premolar was significantly lower than the proportion beyond the mesial end of the upper second premolar (95% CI of 11.2–32.0%, *p* = .00001). There was no significant difference between gender, smoking status, gingival phenotype and palatal shape with the posterior extension of palatal rugae.

**Conclusions:**

Palatal rugae in a sample of a Jordanian population extends beyond the mesial aspect of the upper second premolar which may cause a substantial limitation for graft harvesting from the palate. The hard palate of Jordanian patients may not be a reliable source of soft tissue grafts required for aesthetic mucogingival surgery. No significant association existed between the most posterior extent of palatal rugae and gender, gingival phenotype or palatal shape. Other possible sources should be explored.

## Background

Palatal rugae, known as rugae palatina and plicae palatinae transversae, refer to the elevations located on the anterior part of the palatal mucosa, on either side of the mid palatine raphe and behind the incisive papilla [19]. They comprise of 3–7 ridges, rigid and oblique, that radiate out tangentially from the incisive papilla (just anterior to the underlying incisive foramen) [13–33]. The corrugated, ridge-like structures on the anterior portion of hard palate are conserved in all mammals [22].

Diverse classification methods of palatal rugae exist based on the number, extend, shape and type of the rugae [35]. They serve as a reference landmark in various dental treatment modalities and can be used in the identification of submucosal clefts. They facilitate the transport of food via the oral cavity, participate in crushing food and prevent loss of food from the mouth. In addition, they contribute to taste perception due to the presence of tactile and gustatory receptors. Rugae also contribute to mechanical food qualities and tongue position [35].

Interestingly, palatine rugae can be used as a reliable guide in forensic identification [15] due to the its characteristic uniqueness and high individuality [15–35]. Based on the comparison of the morphological characteristics of palatal rugae, it may have a practical application in the field of personal identification [13–33]. Patterns of palatal rugae have long been studied for various purposes, particularly in the fields of anthropology, genetics, comparative anatomy, orthodontics, forensic odontology and prosthodontics [3–12, 19–34, 38]. In the field of orthodontics, it can also be used to assess the amount of anteroposterior tooth movement because they remain stable during a person’s life and as such are useful in the field of orthodontics [16–23, 38–42].

The posterior extent of the rugae plays an important role in limiting the anterior extension of the soft tissue graft donor site [9–39]. The hard palate and tuberosity is the preferred site for harvesting full epithelialized free grafts or subepithelial connective tissue grafts for oral and periodontal soft tissue augmentation procedures [4–14, 41]. The procedures include the augmentation of the width of keratinized tissue, treatment of gingival recession over teeth and surgical correction of localized alveolar ridge defects [40–48]. The attached keratinized mucosa palatal to the maxillary premolars is the preferred source of such grafts. If harvested within a soft tissue graft, rugae could cause a persistent esthetic problem because it has the tendency to survive with its distinct shape [9] and if incised from the free gingival graft, they re-establish themselves. This property poses an anatomical barrier since palatal rugae have an unaesthetic appearance and should not be included in free gingival grafts [7–32]. The posterior extent of the palatal rugae may pose important limitations for the hard palate as a potential donor site for soft tissue grafts in mucogingival surgeries. It is important to be knowledgeable about the posterior extent of the palatal rugae as it may limit the anterior extent of the soft tissue palatal graft.

Literature related to the distal extent of the rugae is limited and inadequate despite the importance of such information. Interestingly, rugae patterns vary between ethnicities. Kapali and colleagues [19] studied the pattern of palatal rugae in Australian Aborigines and Caucasians and observed the number, length, shape, direction and unification of the rugae. Several studies have shown a significant association between rugae forms and ethnicity [1, 2, 17–27, 29–31, 37]. Due to the variation of the rugae pattern in different ethnicities, there is a need to provide information on the distal extent of the rugae in the Jordanian population. Existing studies in this population are limited to the characteristics of rugae, individuality and gender specificity [11–28].

The purpose of the research was to investigate the distal extension of the rugae area in a Jordanian (Middle Eastern) population as an anatomical limitation influencing the surgical decision for harvesting a palatal soft tissue graft. In addition, factors that may influence or predict extension were assessed.

## Methods

This cross-sectional study recruited sixty (60) systemically healthy Jordanian participants from patients attending the Periodontics clinic and Oral and Maxillofacial Surgery clinic at the Dental Teaching Center, Jordan University of Science and Technology. The sample size of 60 achieves 80% power to detect a difference (P1-P0) of 0.2 using a two-sided Z-test that uses S(P0) to estimate the standard deviation with a significance level (alpha) of 0.050 and a drop out rate of 20%. These results assume that the population proportion under the null hypothesis (P0) is 0.5000. The sample size was calculated using PASS 2020, v20.0.4

Participants had full maxillary dentition (except for third molars). The participants were scheduled for surgical or non-surgical procedures indicated for anesthesia of the hard palate. The study protocol was approved by the university Institutional Review Board Committee.

The exclusion criteria were as follows: history of any disease or surgery in the palate or tuberosity surgery, presence of any dental appliances in the upper, previous orthodontic treatment, extracted or congenitally missing premolars, medication that would affect the periodontal soft tissue, maxillary posterior teeth malposition or malalignment. Patients who declined participation, were excluded.

After explaining the protocol to the participant, the consent form was signed. The patients sat comfortably on the dental chair and a complete oral examination was done. The backrest of the dental chair was raised to an angle of 45° for the maxillary impression. The dental chair was raised so that the operating area was at the elbow level of the operator. The dentist stood behind the participant. Maxillary perforated metal trays were selected according to the shape and size the patient's arches and tested by checking the extension of the trays in the patient's mouth.

The container of the alginate impression material was shaken vigorously before use to ensure complete mixing of the contents. The manufacturer’s provided powder scoop was used to measure two level scoops of the impression material and mixed with 40 ml water, using a measuring jar provided by the manufacturer in a flexible rubber bowl. The ratio was a water/powder (W/P) ratio of 40 ml: 15 g. A vigorous figure-eight motion was used to mix the contents for 45 s to 1 min and a smooth creamy mixture was obtained. The mix was transferred immediately to the impression tray for insertion into the patient’s mouth. The tray was held passively and motionless during the setting of the impression material. After about 2 min (setting time Alginate), the tray was separated quickly from the teeth. Excess material at the periphery was trimmed. The alginate impression was kept on the mechanical vibrator and the mixed dental stone (W/P ratio of 28 ml: 100 g) was added to the impression in small increments to avoid air entrapment. The cast was separated from the impression after 60 min. For the study, base casts were made using the base former and dental stone. Each cast was numbered for identification.

The posterior extent of the rugae was measured using a standardized probe. All the measurements were taken by a single observer (AAK), in a well illuminated room. The study casts were placed on a horizontal base and each stone cast was evaluated bilaterally. The measurements were highlighted from the origin of the rugae (near mid palatine raphe) to the terminal end with a sharp graphite pencil on the cast and a magnification lens was used for identification. This way the most posterior extension of the rugae were marked on the casts and then analyzed.

The normal approximation test was performed with 95% CI on the random sample of 60 subjects to determine the proportion of the subjects with rugael extensions beyond the mesial end of the upper second premolar. The "rugael extension proximal to the mesial end of the upper 2^nd^ premolar" was considered to be the "success" category. Significant associations with potentially contributing factors including gender and smoking were analyzed with Fisher’s Exact tests. The analysis was performed using statistical software (SPSS®, version 23.0). Statistical hypotheses tests were 2-tailed comparisons and the criteria for statistical significance were accepted at the probability level *p* < 0.05.

## Results

**Fig. 1:**
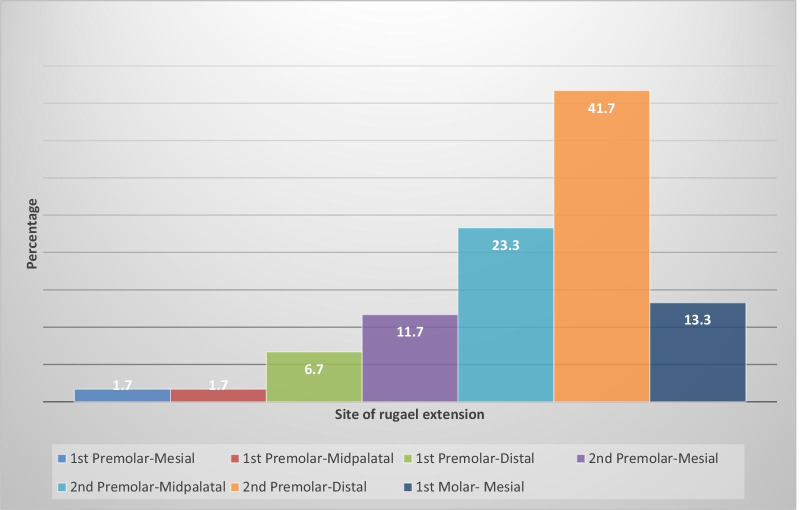
Distribution of rugae distal extension frequencies by site. *Note*: It can be seen that the largest percentage with the highest distal extension frequency is that representing the distal of the second premolar. Most of the rugae extended distally beyond the mesial aspect of the upper second premolar. Rugae extension mesial to the upper first premolar represents only a minute percentage of the total

A total of 60 participants were included in the study. In 41.7% of the participants, the rugae extended distal to the upper second premolar, 23.3% extended to the mid-palatal of the upper second premolar, with 11.7% extending to the mesial of the upper second premolar. The implication is that 90.0% of the rugae reached the upper second premolar and 78.3% extended beyond its mesial aspect (Fig. 1, Table 1). Of the 60 subjects, who were randomly selected, in 13 (22%) the posterior extension of the palatal rugae was proximal to the the mesial aspect of the 2nd premolar and 47 (78%) had the rugae extension beyond the mesial end of the 2nd premolar.

**Table 1:** Percentage distribution of the posterior extension of the palatal rugae in relation to the teeth and according to gender, palatal shape and gingival phenotype

Posterior most extension of the rugae	Percentage (%)	Gender		Palatal shape		Gingival phenotype		Smoking status	
		Male n(%)	Female n(%)	High, narrow n(%)	Shallow, widen(%)	Thin n(%)	Thick n(%)	Smokers n(%)	Non-smokers n(%)
Mesial of 1st premolar	1 (1.7)	1 (1.7)	0 (0)	1 (1.7)	0 (0)	1 (1.7)	0 (0)	0 (0)	1 (1.7)
Midpalatal of 1st premolar	1 (1.7)	0 (0)	1 (1.7)	1 (1.7)	0 (0)	0 (0)	1 (1.7)	0 (0)	1 (1.7)
Distal of 1st premolar	4 (6.7)	0 (0)	4 (6.7)	2 (3.3)	2 (3.3)	2 (3.3)	2 (3.3)	1 (1.7)	3 (5.0)
Mesial of 2nd premolar	7 (11.7)	3 (5.0)	4 (6.7)	6 (10)	1 (1.7)	0 (0)	7 (11.7)	0 (0)	7 (11.7)
Midpalatal of 2nd premolar	14(23.3)	9 (15.0)	5 (8.3)	9 (15)	5 (8.3)	6 (10.0)	8 (13.3)	1 (1.7)	13 (21.7)
Distal of 2nd premolar	25 (41.7)	13 (21.7)	12 (20)	13 (21.7)	12 (20)	9 (15)	16 (26.7)	4 (6.7)	21 (35)
Mesial of 1st molar	8 (13.3)	3 (5.0)	5 (8.3)	6 (10)	2 (3.3)	2 (3.3)	6 (10.0)	0 (0)	8 (13.3)
Total	60 (100)	29 (48.3)	31 (51.7)	38 (63.3)	22 (36.7)	20 (33.3)	40 (66.7)	6 (10)	54 (90)

Note that most of the rugae extend beyond the mesial aspect of the upper second premolar regardless of the gender or palatal shape

The normal approximation test performed with 95% CI with the "rugael extension proximal to the mesial end of the upper 2nd premolar" considered to be the "success" category revealed the proportion for the proximal end had a 95% CI of 11.2–32.0%, *p* = 0.00001.

In terms of the gingival phenotype, the majority (66.7%) consisted of thick, less scalloped gingival phenotypes and 33.3% were thin, more scalloped gingival phenotypes. With regards to rugae extension in the thick phenotype group, 26.7% of the 66.7% ended distal to the upper second molar, 13.3% in its mid-palatal aspect and 10.0% mesial to the first molar. The result is that 61.7% of the 66.7% ended beyond the mesial aspect of the upper second premolar and none ended anterior to the mid-palatal aspect of the upper first premolar. For the thin gingival phenotype, 28.3% of the 33.3% ended beyond the mesial aspect of the upper second premolar and 5% ended proximal to the mesial of the upper second premolar. The Fisher’s exact test showed no significant association between gingival phenotype and the posterior extension of palatal rugae (p = 0.232).

The Fisher’s exact tests revealed no significant association between the posterior extension of the palatal rugae and other factors such as gender (p = 0.245), smoking (p = 0.574) and palatal shape (p = 0.228). The majority (63.3%) of the participants exhibited a narrow, high palate and 36.7% wide, shallow palates.

## Discussion

Several studies investigated the patterns, shape, direction, and unification related to rugae due to the specificity to racial groups and the identification of the population [11–21, 43]. To our knowledge, this was the first study investigating the extension of the rugae relative to the tooth, as an anatomical limitation for harvesting soft tissue grafts through assessing the potential donor site in the Jordanian population. The study also investigated the association between gender, gingival phenotype, palatal shape or smoking and the distal extension of rugae in Jordanians.

Our study revealed that the proportion of the subjects with rugael extensions proximal to the mesial end of the upper second premolar was significantly lower than the proportion beyond the mesial end of the upper second premolar (95% CI of 11.2–32.0%, *p* = 0.00001). Also, no significant associations of the palatal rugael extension with other factors such as smoking, gender, palatal shape and gingival phenotype were found.

Based on literature, the rugae pattern is specific to racial groups. Goria (1911) defined the rugae as the ridges that extend at least one-half the distance from the median palatal raphe to the dental arch [24]. Considering that the occurrence, number and arrangement of the palatal rugae in mammalians are specie-specific [6], racial differences exist. This was demonstrated with twin studies revealing that rugae patterns have an underlying genetic basis [24] and different races. Thomas and Kotze were able to discern different rugae patterns in the South African population, indicating different genetic origins [43–45]. Differences in ethnicity are associated with differences in the pattern and extent of growth of the palate, genetic variation, and different patterns of movement of the teeth due to crowding and the wear pattern. The number of primary rugae and the mean number of rugae in Australian Aborigines was higher than in Caucasians but Caucasians had a higher proportion of rugae longer than 10 mm [19]. As early as 1987, palatal rugae was suggested as a means of an improved statistical method for the racial classification of man [46]. A comparative study of the palatal rugae and shape of the hard palate in Japanese and Indian children indicated no differences between gender but reported that the rugae zone of the left side was shifted further back than the right side. The posterior limit of the rugae zone differed between Japanese and Indian children [21]. In a recent study comparing the palatal rugae pattern in Tibetean and Mysorean populations, a statistically significant association between the total number of rugae and gender was found in both populations. It was also reported that the parameters such as the length and shape of the rugae indicate racial differences [18].

The most appropriate area for graft harvesting is the canine-premolar area, 8–13 mm from the midpalatal aspect of each respective tooth. At this position, there less risk of endangering the greater palatine bundle but with an inherent risk of an aesthetic unacceptable outcome, particularly in free gingival grafts, due to the presence of the rugae [36].

The consequences of transplanting tissue from the anterior palate, which contains rugae, has not been documented extensively. Soehren et al. reported, in a clinical and histological study, only 2 cases of retained rugae in 20 free gingival graft biopsies examined [39], and Breault et al. reported a retained palatal rugae in a free gingival graft 9 years after the surgery, despite the fact that a gingivoplasty was performed 2-months post-surgery. The transplanted rugae remained a permanent part of the recipient site, regardless of the efforts to eliminate them. The group recommended avoidance of these anatomic landmarks when harvesting the graft tissue for esthetic reasons [5]. The characteristics of the epithelium are determined by the underlying connective tissue [9, 10, 20] and the clinical removal of rugae in the palatal donor tissue is not a permanent correction of the topography, since they tend to reappear several months post-treatment, as reported by Coslet et al. and Breault et al. [5–9]. It is only natural that the structural characteristics of the palatal mucosa are conserved as free mature gingival grafts, as reported by Matter et al. [25]. Rateitschak et al. stated that grafts must not contain rugae from the anterior area of the hard palate nor encroach on the soft palate [47]. Cohen recommended that the donor tissue should be harvested from the posterior part of the palate, distal to the anterior rugae as this area contained the widest gingival zone and the least amount of submucosa [8].

Our findings revealed that 90.5% of the rugae reached the upper second premolar and 88.3% extended beyond its mesial aspect. So, basically, the rugae zone extended to the upper second premolar area which poses a problem, as the premolar area in the Jordanian population is the most appropriate donor graft area [36]. The problem is further complicated by the rugae extending as far back as the premolars, not only mesiodistally but also mediolateraly, indicating that it extended very close to the median palatine raphe posteriorly in most of the sample. The rugae that are not in a straight line mediolateraly, extended slightly anterior to the most posterior extent in the central part of the palate (beyond 13 mm from the gingival margin). Taking a soft tissue graft from the area most appropriate for graft harvesting (canine-premolar region) would result in the unaesthetic implantation of palatal rugae.

Our study did not reveal a significant difference with regard to gender, which is contradictory to literature where a moderate discrimination with respect to gender has been identified in the number, length, size and direction of the rugae [11–26]. However, the studies did not take into account the posterior extension.

Although we found no significant difference in the posterior extension of the rugae with regard to smoking status, this has to be interpreted with caution due to the small proportion of smokers (10.3%) in the study. This may be due to no actual association or the small contribution of smokers in the overall sample size.

The strengths of the current study include the exclusion of participants wearing removable appliances, which minimizes the risk of mechanical trauma of the palatal mucosa. The consequence would be minimalizing the potential interference or contribution of confounding factors and other factors that may influence the rugal morphology and the reported outcomes of this clinical study. The majority of the sample (91.4%) was between 15 and 30 years, the age range that is referred for periodontal mucogingival surgery requiring soft tissue graft harvesting from the hard palate. In addition, our study had an equal distribution of male and female and indicated a significant difference between gender and the distal extension of the rugae. A previous study reported significant gender differences in the characteristics of palatal rugae in a Jordanian population, however, the distal extension of the rugae was not investigated [11]. Our major limitation includes the involvement of a single examiner and an inadequate sample size which limits the possibility to extrapolate the results of our study to the broader population.

Finally, evidence from this study indicates, that in the Jordanian hard palate, the presence of rugae presents an anatomic limitation for harvesting soft tissue grafts. However, it is important to note that additional studies with bigger sample sizes are required to generalize our findings to the Jordanian population and to explore the potential risks of harvesting soft tissue grafts from deeper areas in the palate. If the hard palate is not considered adequate for soft tissue graft harvesting in the Jordanian population, other donor areas such as the tuberosity should be considered and investigated. Also, future studies are required to evaluate the use of alternative sources of soft tissue (xenografts and allografts) and guided tissue regeneration to treat mucogingival defects in Jordanian patients.

## Conclusions

Within the limitations of this study, the palatal rugae in most of the Jordanian sample extends beyond the mesial aspect of the upper second premolar and this may cause a severe limitation for graft harvesting from the palate. A possible site for harvesting thick soft tissue grafts of 2 mm in the Jordanian hard palate is the canine-premolar region, 8–13 mm from the gingival margin. The presence of rugae poses a limitation for graft harvest. Overall, the hard palate of Jordanian patients does not represent a reliable source of soft tissue grafts required for esthetic mucogingival surgery. No significant correlation existed between the most posterior extent of palatal rugae and gender, age, gingival phenotype or jaw type. Other possible sources should be explored.

## Data Availability

The datasets used and analysed during the current study are available from the corresponding author on reasonable request.

## Abbreviations

W/P: Water/powder
P0 = P1: P0 is the value of the population proportion under the null hypothesis
P0 ≠ P1: P1 is the value of the population proportion under the alternative hypothesis
P1-P0: Difference to be detected by the study
